# Adjusting for overdispersion in piecewise exponential regression models to estimate excess mortality rate in population-based research

**DOI:** 10.1186/s12874-016-0234-z

**Published:** 2016-10-01

**Authors:** Miguel Angel Luque-Fernandez, Aurélien Belot, Manuela Quaresma, Camille Maringe, Michel P. Coleman, Bernard Rachet

**Affiliations:** Department of Non-Communicable Disease Epidemiology, Faculty of Epidemiology and Population Health, London School of Hygiene and Tropical Medicine, Cancer Survival Group, Keppel Street, London, WC1E 7HT UK

**Keywords:** Epidemiologic methods, Regression analysis, Survival analysis, Proportional hazard models, Cancer

## Abstract

**Background:**

In population-based cancer research, piecewise exponential regression models are used to derive adjusted estimates of excess mortality due to cancer using the Poisson generalized linear modelling framework. However, the assumption that the conditional mean and variance of the rate parameter given the set of covariates *x*_*i*_ are equal is strong and may fail to account for overdispersion given the variability of the rate parameter (the variance exceeds the mean). Using an empirical example, we aimed to describe simple methods to test and correct for overdispersion.

**Methods:**

We used a regression-based score test for overdispersion under the relative survival framework and proposed different approaches to correct for overdispersion including a quasi-likelihood, robust standard errors estimation, negative binomial regression and flexible piecewise modelling.

**Results:**

All piecewise exponential regression models showed the presence of significant inherent overdispersion (*p*-value <0.001). However, the flexible piecewise exponential model showed the smallest overdispersion parameter (3.2 versus 21.3) for non-flexible piecewise exponential models.

**Conclusion:**

We showed that there were no major differences between methods. However, using a flexible piecewise regression modelling, with either a quasi-likelihood or robust standard errors, was the best approach as it deals with both, overdispersion due to model misspecification and true or inherent overdispersion.

**Electronic supplementary material:**

The online version of this article (doi:10.1186/s12874-016-0234-z) contains supplementary material, which is available to authorized users.

## Background

In population-based cancer research, the relative survival setting is used because the cause of death is often not available or considered to be unreliable [1]. Therefore, the survival and the mortality associated with cancer are estimated by incorporating the information of the expected mortality from the general population (i.e. background mortality) obtained from national or regional life tables [1, 2]. The main advantage of the relative survival setting is that it provides a measure of patients survival and mortality associated with cancer without the need for information on the specific cause of death [1]. These measures of survival and mortality are known as the net survival and the excess mortality respectively [2–4]. When multivariable adjustment is of interest, the excess mortality can be modelled using piecewise exponential regression models [3, 5]. Piecewise exponential regression excess mortality (PEREM) models derive adjusted excess mortality rates accounting for the expected mortality of the background population [5, 6].

It has been shown that PEREM models can be fitted in the Generalized Linear Modelling (GLM) framework [3]. Using the GLM framework it is relatively easy to extend the models to deal with clustering, through either a random-effects model or by utilizing sandwich-type estimators for the standard errors (SE) [6–8]. To fit PEREM models follow-up time is split into k intervals (e.g., yearly, monthly) and person-times of follow-up *y*_*k*_ is introduced as an offset in the model, assuming that the excess mortality rate is constant within each interval but, it can vary arbitrarily between the intervals. Moreover, the usual assumption that the number of deaths (*d*_*k*_) observed in interval k can be described by a Poisson distribution with rate parameter $\lambda _{k}\,=\,\frac {d_{k}}{y_{k}}$ has been adapted to the relative survival setting [3].

The rate parameter *λ*_*k*_ is adapted to include the expected mortality of the general population under the relative survival setting

1 $$ \lambda_{k}^{+}\,=\,\frac{d_{k}-d_{k}^{*}}{y_{k}}\,=\,\frac{d^{+}_{k}}{y_{k}},  $$

where (*d*_*k*_) and ($d^{*}_{k}$) are the observed and expected number of deaths from the general population and $\left (d^{+}_{k}\right)$, the excess number of deaths.

Thus, the Log-likelihood for the PEREM model includes the updated rate parameter:

2 $$ ln\left(\lambda_{k}^{+}\right) = ln\left(\lambda^{+}_{0k}\right)\,+\,\mathbf{x}^{\mathrm{T}}\boldsymbol{\beta},  $$

where $ln(\lambda _{k}^{+})$ is the logarithm of the excess mortality and **x** ^T^ denotes the transpose of the vector of covariates **x** and ***β*** represent the corresponding parameter estimates.

Using (1), we can rewrite the rate parameter defined in (2) as:

3 $$ ln\left(d_{k}-d_{k}^{*}\right) = ln(y_{k})\,+\,ln\left(\lambda^{+}_{0k}\right)\,+\,\mathbf{x}^{\mathrm{T}}\boldsymbol{\beta},  $$

where *l* *n*(*y*_*k*_) is the logarithm of the person-time at risk for the *k* ^*th*^ interval incorporated in the model as an offset and $ln(\lambda ^{+}_{0k}$), is the log of the baseline excess mortality rate [3, 6].

Using (1), we can rewrite the PEREM model in (3):

$$\frac{d_{k}}{y_{k}}\,=\,\frac{d^{*}_{k}}{y_{k}}\,+\,\lambda^{+}_{0k}\,\exp\left(\mathbf{x}^{\mathrm{T}}\boldsymbol{\beta}\right). $$

The model in (3) assumes constant rates over intervals of time and it may lead to overdispersion due to extra variability in the rate parameter (i.e. the variance is greater than the mean). The assumption that the conditional mean and variance of the rate parameter given covariates **x** are equal is strong and may fail to account for inherent or genuine overdispersion. The variance exceeds the mean generally because of positive correlation between variables or excess variation between rates [9].

Overdispersion in PEREM models is typically due to extra variability in the rate parameter (genuine overdispersion). However, other forms of non-genuine overdispersion may appear when the model omits important explanatory predictors; the data contain outliers, or the model fails to introduce enough interaction terms or non-linear functional form between predictors and outcome. By contrast, no external remedy can be applied in the case of inherent or genuine overdispersion [10].

Fitting an overdispersed PEREM model leads to underestimating standard errors (SE) and therefore to the inappropriate interpretation of the conditional estimates of the covariates introduced in the model (i.e. a variable may be wrongly considered as a significant predictor).

Using an empirical example, we aim to take advantage of the relationship between the GLM framework and the PEREM model to apply a simple method to test and correct for overdispersion that could be easily implemented and used by population-based cancer researchers.

## Methods

The presence of overdispersion can be recognized when the value of the ratio between the Pearson *χ* ^2^ (or deviance statistics) over the degrees of freedom is larger than one. However, a more formal statistical approach is required to test the presence of inherent overdispersion, then to correct for it [11].

### Testing overdispersion in PEREM models

A regression-based score test enables us to evaluate whether the variance is equal to the mean (*V* *a* *r*(*λ* ^+^) = *E*(*λ* ^+^)) or proportional to the square mean [11]:

4 $$ Var\left(\lambda^{+}\right)\,=\,E\left(\lambda^{+}\right)\,+\,\alpha\,E\left(\lambda^{+}\right)^{2},  $$

We first calculate the score statistic (Z) to test H0: *α*=0 against H1: *α* > 0, using the fitted values of the excess mortality rate $\widehat {\lambda ^{+}}$ [11–13]:

$$Z\,=\,\displaystyle\sum_{i=1}^{N}\displaystyle\sum_{k=1}^{M}\left(\frac{\left(\lambda^{+}_{ik}-\widehat{\lambda_{ik}^{+}}\right)^{2}\,-\,\lambda^{+}_{ik}}{\widehat{\lambda_{ik}^{+}}}\right), $$

where $\lambda ^{+}_{ik}\,=\,\frac {d^{+}_{ik}}{y_{ik}}$ and substituting *λ*_*ik*_ and $\widehat {\lambda _{ik}^{+}}$ gives:

5 $$ Z\,=\,\displaystyle\sum_{i=1}^{N}\displaystyle\sum_{k=1}^{M}\left(\frac{\left(\frac{d^{+}_{ik}}{y_{ik}}\,-\, \frac{\widehat{d^{+}_{ik}}}{y_{ik}}\right)^{2}\,-\,\frac{d^{+}_{ik}}{y_{ik}}}{\frac{\widehat{d^{+}_{ik}}}{y_{ik}}}\right).  $$

The test is implemented by a linear regression of the generated dependent variable Z on $\widehat {\lambda _{ik}^{+}}$ (independent variable), without including an intercept term. Hence the output can be interpreted as a *T*-test of whether the coefficient of $\widehat {\lambda _{ik}^{+}}$ is zero testing whether the variance of the rate parameter is equal to the mean [12].

### Correcting for overdispersion

The most commonly used approaches to correct for inherent overdispersion are relatively straightforward to implement in common statistical software.

#### Quasi-likelihood approach

Inherent overdispersion in PEREM modeling may be due to extra variability in the parameter $\lambda _{ik}^{+}\,=\,\frac {d_{ik}-d_{ik}^{*}}{y_{ik}}$. Including an extra parameter *ϕ* in the model allows the variance to vary freely from the mean [14]. There are several options to compute the extra parameter *ϕ*. The simplest is to take *f*(*λ* ^+^,*ϕ*) = *ϕ*×*λ* ^+^, which specifies a constant proportional overdispersion *ϕ* across all individuals. Using a PEREM modeling approach, we assume that the distribution of $\lambda _{ik}^{+}$ is Poisson. Hence the Pearson Chi-squared statistic can be computed as a criteria of goodness of fit using the observed (O) and expected values (E) from the model:

$$\chi^{2}\,=\,\displaystyle\sum_{i=1}^{n}\frac{(O_{i}-E_{i})^{2}}{E_{i}}. $$

Substituting O and E by $\lambda ^{+}_{ik}$ and $\widehat {\lambda _{ik}^{+}}$ gives:

$$\chi^{2}\,=\,\displaystyle\sum_{i=1}^{N}\displaystyle\sum_{k=1}^{M}\left(\frac{\left(\frac{d^{+}_{ik}}{y_{ik}}\,-\, \frac{\widehat{d^{+}_{ik}}}{y_{ik}}\right)^{2}}{\frac{\widehat{d^{+}_{ik}}}{y_{ik}}}\right). $$

The ratio between the Pearson *χ* ^2^ or deviance statistic, and the degrees of freedom should be closed to one as we expect that the variance of the model is equal to that of the assumed Poisson distribution. We can estimate the overdispersion parameter *ϕ* multiplying the inverse of the degrees of freedom (df) of the model times the Pearson *χ* ^2^ statistic.

Scaling the SE with $\sqrt {\hat \phi }\,=\,\sqrt {\chi ^{2}/df}$ will correct the estimated SE of $\hat {\beta }$, which was estimated under the model of constant overdispersion [15, 16]. The estimated $\hat \phi $ is integrated as a scalar updating the variance-covariance matrix of the PEREM model estimated under the GLM framework and thus correcting for overdispersion [17]. Under the GLM framework $\hat {\beta }$ and the SE of $\hat {\beta }$ is optimized via an iteratively reweighted least squares procedure [11, 17]. Therefore, scaling the SE of $\widehat {\beta }$ in terms of matrix notation is given by [17]

$$\text{Variance}(\hat{\beta})\,=\,\hat{\phi}\,\left(\mathbf{X}^{T}\mathbf{WX}\right)^{-1}, $$

where **X** represents the n ×p design matrix of the observed data and, **W** is a diagonal n ×n matrix with the values of $\widehat {\lambda ^{+}}=\exp (\mathbf {x}^{\mathrm {T}}\boldsymbol {\beta })$ on the diagonal. Thus, the variance is updated with the new values of the weighted matrix under the assumption of no specific probability distribution [14].

#### Robust standard errors of parameters estimates

In maximum likelihood estimation, the standard errors of the estimated parameters are derived from the Hessian (matrix of second derivatives on the parameters) of the likelihood. However, theses standard errors are correct only if the likelihood is the true likelihood of the data [14]. In cases where we consider that overdispersion might be due to unobserved covariates and the link function or the probability distribution function are misspecified, the assumption about the true likelihood of the data does not hold. Under these scenarios, we can still use robust estimates of the standard error known as Huber, White, or sandwich variance estimates to correct for overdispersion Additional file 1 [18–20].

$$\text{Variance}(\hat{\beta})\,=\,(\mathbf{X}^{T}\mathbf{WX})^{-1}(\mathbf{X}^{T}\mathbf{\Sigma X})(\mathbf{X}^{T}\mathbf{WX})^{-1}, $$

where **Σ** is a n ×n matrix with the values of $(\lambda ^{+}\,-\,\widehat {\lambda ^{+}})^{2}$ on the diagonal.

#### Negative binomial regression model

Given the presence of heterogeneity of subject-specific rates leads naturally to the question of whether we can model subject-specific rates using a random effects framework. The simplest random effects model assumes a person-to-person heterogeneity can be expressed by a model for the mean along with a log-Gamma distribution of the random intercept term. The random intercept follows a log-Gamma distribution, and the marginal distribution of the outcome followed a negative binomial distribution which has two parameters, shape (*E*(*λ* ^+^)) and scale (*V* *a* *r*(*λ* ^+^)), but importantly its variance and mean are related by (4), where the parameter *α* must be positive allowing the variance of *λ* ^+^ to be greater than the mean [11]. The Poisson distribution is a special case of the negative binomial distribution where *α* = 0 [11]. We can estimate *α* using the coefficient from a linear regression with Z (5) as dependent variable on $\widehat {\lambda ^{+}}$ (independent variable), without including an intercept term, as described above [12].

#### Flexible PEREM model

The piecewise exponential regression model under the GLM and relative survival frameworks could be extended by finely splitting the time scale and using a flexible function of time such as splines [21, 22]. The flexible PEREM models allow modelling the baseline hazard and any time-dependent effects as smooth and continuous functions [6]. A time-dependent effect is easily modelled by including an interaction term between the smooth function of time and the covariate [23]. Cubic regression splines is a very popular choice for modelling flexible functions. In a truncated power basis, a cubic regression spline s(t) of time t, with K knots located in different places of the distribution of the smooth function of time can be written as [5]:

$$s(t)\,=\,\sum_{j=0}^{3}\,\beta_{0j}t^{j}\,+\,\sum_{i=1}^{K}\beta_{i3}(t\,-\,k_{i})^{3}_{+}, $$

where

$$(\text{t} \,-\,k_{i})^{3}_{+}\,=\, \left\{\begin{array}{ll} (\text{t} \,-\, k_{i})^{3} & \quad \text{if t} > k_{i},\\ 0 & \quad \text{otherwise}.\\ \end{array}\right. $$

In order to deal with high variance at the outer range of the predictors, they may be forced (restricted) to be linear before the first knot and after the last knot leading to a natural or restricted cubic spline [23]. The first and the last knots are known as boundary knots [24, 25]. If we define m interior knots, *k*_1_,…,*k*_*m*_, and also two boundary knots, *k*_*min*_,…,*k*_*max*_, we can now write *s*(*t*) as a function of parameters *γ* and some newly created variables *z*_1_,…,*z*_*m*+1_, giving [5]:

$$s(t)\,=\, \gamma_{0}\,+\,\gamma_{1}z_{1}\,+\,\gamma_{2}z_{2}\,+\,\ldots\,+\,\gamma_{m+1}z_{m+1}, $$

The basic functions *z*_*j*_(*j*=2,…,*m*+1) are derived as follows:

$$\begin{array}{*{20}l} {}z_{1}\,&=\,t,\\ {}z_{j}\,&=\,(x - k_{j})^{3}_{+} - \lambda_{j}(x - k_{min})^{3}_{+} - \left(1 - \lambda_{j}\right)(x - k_{max})^{3}_{+},\\ {}\lambda_{j}\,&=\,\frac{k_{max} - k_{j}}{k_{max} - k_{min}}. \end{array} $$

These functions can be easily implemented using various Stata commands (e.g., rcsgen) [5, 6]. The flexible PEREM approach using splines allows modelling easily non-proportional excess mortality rate ratios including time-dependent effects of the covariates. Thus, we can achieve a better model specification which should minimize non-genuine overdispersion [25]. However, we can still scale the SE estimates in case of inherent overdispersion previously detected using the suggested regression based Score test.

### Illustration

Data were obtained from the Office for National Statistics (ONS), comprising 376,791 women diagnosed with breast cancer in England between 1997 and 2005, with a follow-up to the end of 2012. The event of interest is death from any cause, with follow-up restricted to 7 years after diagnosis though we estimated up to 5 years excess mortality [21]. We built life tables from England to derive the expected mortality in the background population, by sex, single year of age, calendar year, and deprivation quintile. We aimed to estimate excess mortality hazard rate for age and deprivation in the first five years after the diagnosis of a breast cancer. Legal authority to hold the cancer data derives from a contract with the ONS to produce the official national statistics on cancer survival.

### Statistical methods

First, we split the times-to-event to merge the cancer data with the estimated expected number of deaths for all causes using life tables from England [26]. Then, we fitted four types of PEREM models: in model A, we did not correct for overdispersion, in model B we scaled the SEs by the $\sqrt {{\vphantom {\frac {3}{2}}}\widehat {\phi }}$, in model C we used the Sandwich estimates of the SEs and in model D we fitted a NBR assuming a log-gamma distribution. All models were within the GLM framework with the Poisson family and the modified link $\left (ln\left (d_{ik}\,-\,d^{*}_{ik}\right)\right)$. The modified link log was used to incorporate in the maximum likelihood estimation the expected number of deaths (*d* ^∗^) from the background population [3, 5].

Deprivation was included in all PEREM models as a categorical variable, with Q1, the least deprived group, as the reference category. Age was included as a categorical variable with five levels (<50, 50-59, 60-69, 70-79, ≥80) using <50 as reference. Follow-up time was parameterized as a categorical variable in PEREM models and as a smooth function of time for the flexible PEREM models. We reported $\widehat {\beta }$, $\text {var}(\hat \beta)$, and the relative loss in efficiency (RLE) of $\text {var}(\widehat \beta)$. To estimate RLE for each PEREM model corrected for overdispersion, the model not corrected for overdispersion was the reference [27]. The RLE was computed as the ratio between the variance of the estimates from the models adjusted for overdispersion and the variance from the uncorrected model

6 $$ \text{RLE}(\text{var}(\hat{\beta_{2}}),\text{var}(\hat{\beta_{1}}))\,=\,\frac{\text{var}(\hat{\beta}_{2})}{\text{var}(\hat{\beta}_{1})},  $$

where var($\hat {\beta _{2}}$) refers to the corrected estimate of the variance for overdispersion (scaling the SE or using the sandwich robust estimates) and var($\hat {\beta _{1}}$) to the uncorrected. The RLE was interpreted as the percentage of efficiency loss (% of increase in the variance estimate) for PEREM models needed to reduce bias after correction for overdispersion.

Finally, we fitted a flexible PEREM model which included an interaction between deprivation quintiles and follow-up time to allow the effect of deprivation to vary over time. Hence, the baseline rate was defined as a restricted cubic spline, with one-month intervals and five knots placed at the minimum and the maximum and at the 25th, 50th, and 75th centiles of the event times. For this flexible PEREM model, we plotted the excess mortality rate ratios and 95 % CI for each quintile of deprivation in categories with corrected and uncorrected SE for overdispersion [6, 23, 28]. All analysis were performed using Stata v.14 (StataCorp LP, College Station, Texas, US) Additional file 2.

## Results

**Fig. 1:**
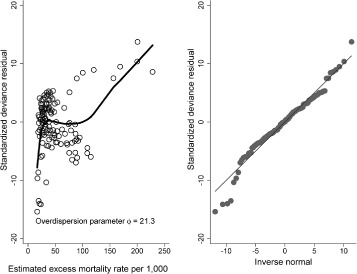
Piecewise exponential regression excess mortality model: standardized Pearson *χ* ^2^ residual analysis, *n*= 376,791 women diagnosed with breast cancer in England between 1997 and the end of 2005

The Pearson *χ* ^2^ deviance residuals were non-normally distributed for the uncorrected PEREM model (Shapiro-Wilk test for normality *p*-value = 0.01) [29], and the overdispersion parameter (*ϕ*) was 21.3 % times higher than expected suggesting the presence of overdispersion. The Score test for overdispersion rejected the *H*_0_ (*p*-value <0.001) indicating the presence of truly overdispersion in the rate parameter and, the scatter plot of the standardized Pearson’s *χ* ^2^ residuals against the excess mortality rates suggested the presence of heteroscedasticity and hence, potential overdispersion (Fig. 1).

Table 1 contrasts the exponentiated coefficients, SE, and RLE for the four different PEREM models, uncorrected (model A) or corrected for the presence of inherent overdispersion (models B, C with *ϕ* parameter = 21.3) and model D adjusted for overdispersion using the NBR approach.

**Table 1:** Piecewise exponential regression excess mortality models with and without correcting for overdispesion, *n* = 376,791 women diagnosed with breast cancer in England between 1997 and the end of 2005

	PEREM A		PEREM B (scaled SE)			PEREM C (Robust SE)			PEREM D (NBR)		
Age at diagnosis	EMRR	SE	EMRR	SE	RLE (%)	EMRR	SE	RLE (%)	EMRR	SE	RLE (%)
50−59*v* *s*.<50	0.75	0.0107	0.75*	0.0493	21.3579	0.75*	0.0576	29.2222	0.75*	0.0380	12.6944
60-69 vs. <50	0.88	0.0130	0.88*	0.0600	21.3580	0.88*	0.0599	21.2823	0.87*	0.0486	14.0296
70-79 vs. <50	1.71	0.0235	1.71	0.1086	21.3578	1.71	0.1324	31.7953	1.65	0.1005	18.3181
≥80 vs. <50	3.39	0.0465	3.39	0.2150	21.3579	3.39	0.3159	46.1198	3.15	0.2222	22.8188
Quintiles of deprivation											
Q2 vs. Q1	1.05	0.0153	1.05*	0.0705	21.3659	1.05*	0.0747	24.0197	1.05*	0.0626	16.8745
Q3 vs. Q1	1.16	0.0166	1.16	0.0767	21.3711	1.16*	0.0873	27.6612	1.15	0.0687	17.1404
Q4 vs. Q1	1.27	0.0182	1.27	0.0839	21.2723	1.27	0.0934	26.3313	1.27	0.0762	17.5240
Q5 vs. Q1	1.48	0.0218	1.48	0.1007	21.3249	1.48	0.1046	23.0039	1.47	0.0885	16.4928

*EMRR* Excess mortality rate ratio, *NBR* Negative binomial regression, *PEREM* Piecewise exponential regression excess mortality model, *RLE* Relative loss in efficiency, *SE* Standard error, *p-value >0.05

The model with the less conservative SE, model A, showed significant excess mortality rate ratio for each of the four deprivation quintiles (compared with the first quintile). Models corrected for overdispersion (B, C, and D) provided more conservative estimates of the SEs. After accounting for oversdispersion, deprivation showed a significant excess mortality, compared to Q1, only for the deprivation quintiles Q3-Q5 for models B and D, and for the deprivation quintiles Q4 and Q5 for model C (Table 1). Compared with the unadjusted model A, all corrected models showed a non-significant effect for age groups 60-69 and 50-59. Overall, the RLE ranged between 12 and 46 percent for corrected models compared with the model uncorrected for overdispersion. The RLE was, however, larger for model C (robust SE). The model D (NBR), compared with model B (scaled SE) and C, showed the smallest RLE. The loss of precision in the models corrected for overdispersion was reflected by the loss of statistical significance for the age groups 50-59, 60-69 and the deprivation quintiles Q1 and Q2. However, scaling the SE to control for overdispersion (model B) showed better efficiency (smaller RLE) compared with the robust SE estimation (model C)(Table 1).

**Fig. 2:**
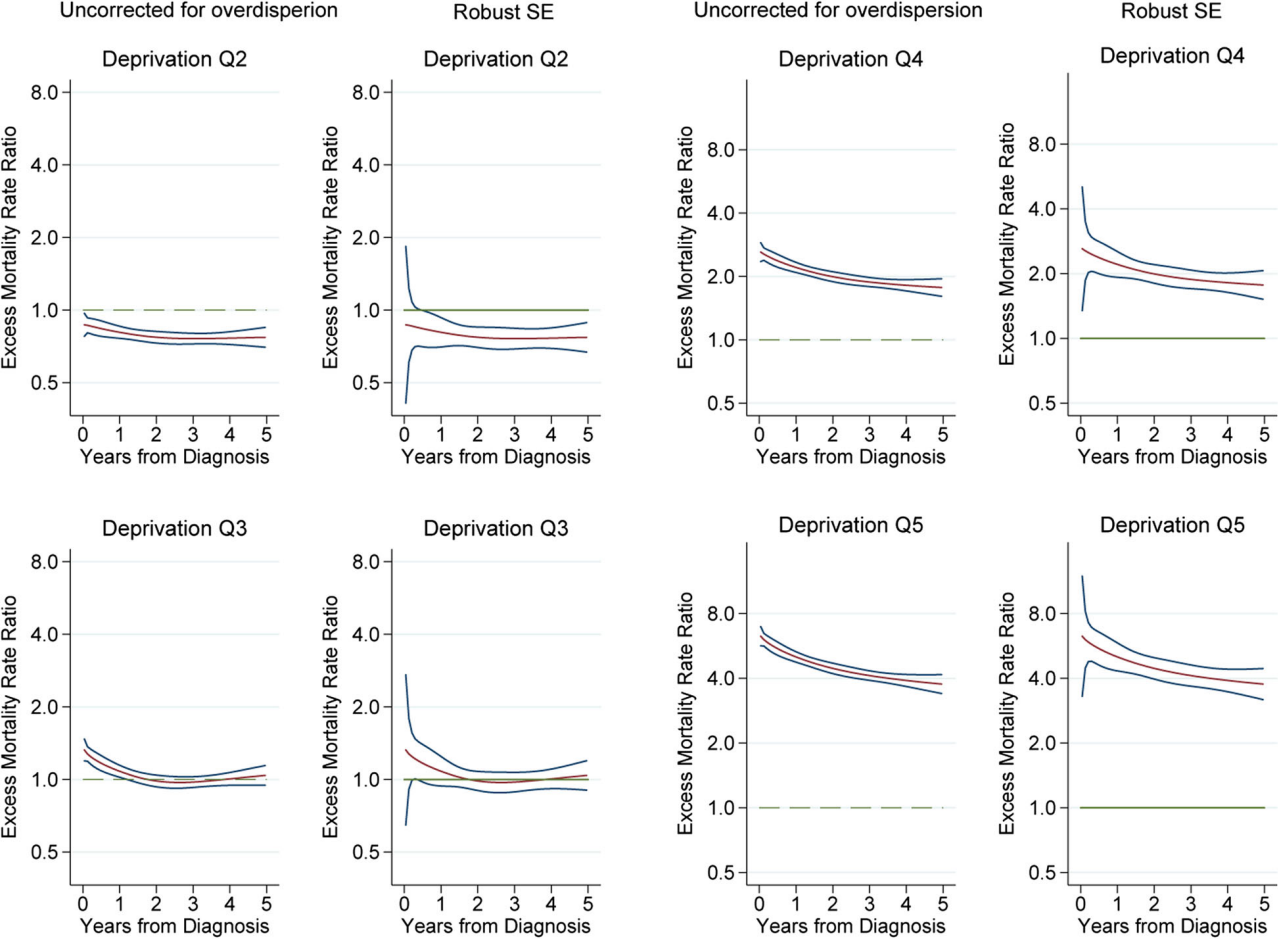
Flexible piecewise exponential regression model: A (non-scaled SE) B (robust SE), *n* = 376,791 women diagnosed with breast cancer in England between 1997 and the end of 2005

Finally, the flexible PEREM model showed smaller overdispersion parameter (*ϕ* = 3.2). The test for overdispersion showed the presence of significant inherent overdispersion (*p*-value <0.001). The flexible PEREM model reduced significantly the overdispersion parameter compared with the models without the smooth functions of time (21.3 vs. 3.2). Allowing for the time dependent effect of deprivation, revealed a decreasing trend of the excess mortality over time during the first five years after the diagnosis of breast cancer. Furthermore, the interaction between the smooth function of time with deprivation showed a stronger effect of deprivation over time, illustrated with 8 to 4 times higher excess mortality rate ratios for the most deprived group compared with the least deprived (Fig. 2).

## Discussion

We have shown that under the relative survival and GLM frameworks, the modified link to fit PEREM models, allows the inclusion in the maximum likelihood estimation of the information regarding the background mortality of the reference population [3]. However, data analysts may expect to find inherent overdispersion as a characteristic of this modelling approach [30].

We have shown, that inappropriate imposition of the Poisson restriction may produce spuriously small SEs of the estimated coefficients $\hat {\beta }$. Fitting an overdisperse PEREM model under the relative survival and GLM frameworks, may lead to underestimate SEs and, therefore, to inappropriate statistical interpretation of the significance of the conditional estimates from the effects of the covariates introduced in the model (i.e., a variable or the levels of a categorical variable, may appear to be significant predictors of the outcome, when in fact it is not).

We encourage epidemiologist and applied statistician using PEREM models under the relative survival framework, to consider to test the Poisson restriction and to relax it, if appropriate, using the methodological approaches described in this article. However, in addition to cancer, the same advice may apply to any other chronic disease or condition for which estimates of disease-specific population-based survival time controlling for competing risk are of interest. We have shown, that using a simple test for overdispersion we can identify significant inherent overdispersion, and applying a pseudo-likelihood estimation, fitting an NBR or a more advanced flexible PEREM modeling approach we can correct for it. These simple approaches may allow applied researchers in population-based cancer registries to infer correct conclusions from the analysis of their data in the presence of significant overdispersion. Applied researchers will have to consider the trade-off between modelling complexity and model interpretation as it might happen that there is no reason for applying a more advanced flexible PEREM modelling given a non-significant overdispersion test. However, it rarely happens, as under the relative survival framework we may expect the presence of overdispersion due to the variability of the rate parameter. Furthermore, in case of a significant overdispersion test, applied researchers will have to consider the compromise of model efficiency (i.e., the precision of the SE) while deciding which method or approach to use to deal with overdispersion. As suggested in our results, the flexible PEREM model showed the smaller loss in precision.

We suggest scaling the SE to correct for overdispersion due to the variability of the rate parameter with a significant overdispersion test and a small overdispersed parameter *ϕ*. However, it should be noticed that our results regarding the RLE are based on only one empirical data. Hence, further investigation is warranted using simulations.

The maximum likelihood methods are based on strong distributional assumptions, while quasi-likelihood or maximum likelihood methods with robust SEs rely on weaker assumptions. Furthermore, using a flexible parametric approach including time-dependent effects allows for a better model specification decreasing overdispersion. We suggest testing for the presence of inherent overdispersion in the data and correct for it using any of the approaches presented in this article. Given that there are no major differences between the above-described methods, the question is not which method to use (robust SE, scaled SE or NBR) in the presence of inherent overdispersed data, but to use any of them to correct for overdispersion and to infer correct conclusions from the models. However, we have shown the benefits of using the flexible PEREM modelling approach with either scaled, robust SE or NBR, under the GLM and relative survival frameworks. Flexible PEREM modelling benefits are double as it deals with model misspecification and overdispersion. The introduction of smooth functions of time and time-dependent effects in the flexible PEREM models may improve the model specification reducing significantly the overdispersion parameter.

## Conclusion

In population-based cancer research, PEREM models are used to estimate the excess mortality rate from cancer under the relative survival framework. We have shown the impact of overdispersion on the excess mortality rate estimates by deprivation among women diagnosed with breast cancer in England between 1997 and the end of 2005. PEREM models are fitted under the assumption of a Poisson distribution leading to overdispersion. We have shown that inappropriate imposition of the Poisson restriction may produce spuriously small estimated standard errors, and thus, wrong interpretation of the model estimates. Given the public health relevance of population-based data analyses for policy and decision making, it is desirable to test for overdispersion and to correct it if appropriate.

## Additional files

Additional file 1 Robust standard error estimation for generalized linear models. (PDF 104 kb)

Additional file 2 Stata do file with commented syntax. (PDF 40 kb)

## Abbreviations

E: Expected value
GLM: Generalized linear model
NBR: Negative binomial regression
O: Observed value
ONS: Office of national statistics
PEREM: Piecewise exponential regression excess mortality model
RLE: Relative loos of efficiency
SE: Standard error
