# Supplementary material for: Adjusting for overdispersion in piecewise exponential regression models to estimate excess mortality rate in population-based research
Source: BMC Med Res Methodol. 2016 Oct 1;16:129. doi: 10.1186/s12874-016-0234-z (PMC5045632; doi:10.1186/s12874-016-0234-z)
Supplement: Additional file 1 — Robust standard error estimation for generalized linear models. (PDF 104 kb) [file 12874_2016_234_MOESM1_ESM.pdf]

Additional file One

Robust standard error estimation for generalized linear models

Let $\theta \in R^p$ be a p x 1 parameter vector and f_i be a positive density function $y_i \sim, f_i(y_i|\theta)$. The data (y_i) are modeled as observed values of Y_i for i = 1,...,n and the likelihood function is given by

$$L(\theta) = \prod_{i=1}^{n} f_i(y_i|\theta),$$

and the log-likelihood function by

$$LL(\theta) = \sum_{i=1}^{n} ln\, f_i(y_i|\theta).$$

First derivative (gradient) of the log-likelihood function

$$L'(\theta) = \sum_{i=1}^{n} g_i(y_i|\theta) = \sum_{i=1}^{n} \frac{\partial ln f_i(y_i|\theta)}{\partial\theta}.$$

Second derivative (hessian) of the log-likelihood function

$$L''(\theta) = \sum_{i=1}^{n} h_i(y_i|\theta) = \sum_{i=1}^{n} \frac{\partial^2 ln f_i(y_i|\theta)}{\partial\theta^2}.$$

Assuming that the model is correct, there is a true value θ_0 for θ. Then, we can use the Taylor approximation of second order for the log-likelihood function to estimate θ.

$$L(\theta) = L(\theta_0) + L'(\theta_0)(\theta - \theta_0) + \frac{1}{2}(\theta - \theta_0)^T L''(\theta_0)(\theta - \theta_0) + \ldots$$

Therefore to derive the variance covariance matrix from the maximum likelihood estimation we can differentiate this expression to get

$$L'(\theta_0) + (\theta - \theta_0)^T L''(\theta_0) = 0.$$

So, $\widehat{\theta} - \theta_0 = [-L''(\theta_0)]^{-1} L'(\theta_0)^T$,

$$Cov(\widehat{\theta}) = [-L''(\theta_0)]^{-1} [Cov(L'(\theta_0)][-L''(\theta_0)]^{-1}$$
