# Supplementary material for: Adjusting for overdispersion in piecewise exponential regression models to estimate excess mortality rate in population-based research
Source: BMC Med Res Methodol. 2016 Oct 1;16:129. doi: 10.1186/s12874-016-0234-z (PMC5045632; doi:10.1186/s12874-016-0234-z)
Supplement: Additional file 2 — Stata do file with commented syntax. (PDF 40 kb) [file 12874_2016_234_MOESM2_ESM.pdf]

Additional file two

```
**********************************************************************
*Controlling for Overdispersion in Piecewise Exponential Excess Mortality
*Regression Models in Population-based Cancer Research
**********************************************************************
****************************************************************************************************
*A) Piecewise Exponential Regression Excess Mortality Models with a without accounting for overdispersion
****************************************************************************************************
use England_breast_1997_2007.dta,clear
stset time, fail(dead=1) exit(time 10) id(ID2)
//Using strs to merge data with background expected number of deaths and mortality rate from England life tables
    strs using popmort_England_2, ///
breaks(0(1)5) noshow ///
diagage(agediag) diagyear(yeardiag) ///
attage(age) attyear(year) ///
mergeby(ctry dep region sex year  age) ///
by(agegroup dep) ///
survprob(survprob) ///
notables savgroup(group.dta, replace)
//Proportional excess hazards model: piecewise exponential regression excess mortality model
use group.dta, clear
quietly tabulate agegroup, generate(agep)
quietly tabulate dep, gen(depr)
glm d ibn.end agep2-agep5 depr2-depr5, family(poisson) link(rs d_star) lnoffset(y) eform nocons nolog baselevels
*Pearson statistic
predict double pred, mu
gen  per = (d-pred)^2/(pred)
egen pearson=sum(per)
sum pearson
// Assessing overdispersion: Analysis of Pearson and Deviance residual & Figure 1
*Expected excess mortality
sum nu
gen double erate= ((d-d_star)/y)
corr nu erate
replace erate=erate*1000
tw line erate midtime if agep5==1 & depr5==1 || line erate midtime if agep5==1 & depr1==1
*Standardized Deviance
predict dev, deviance
*Deviance residuals
predict stdev, standardized deviance
*Goodness of fit and checking model assumptions
lowess stdev erate, scheme(s1manual) xtitle("Expected excess mortality rate per 1,000") plotregion(style(none)), name(Figure1A)
qnorm stdev, scheme(s1manual) xtitle("Inverse normal") plotregion(style(none)), name(Figure1B)
swilk dev
graph combine Figure1A.gph Figure1B.gph, name(Figure1)
//Testing for overdispersion: Score based t-test
glm d ibn.end agep2-agep5 depr2-depr5, family(poisson) link(rs d_star) lnoffset(y) eform nocons nolog baselevels
predict double mu, mu
//Score based t-test: H0 E(x|y) = Var(x|y)
gen double zrs =((d-mu)^2-d)/(mu)
reg zrs mu, nocons nohead
//Negative binomial model Var(x|y) = E(x|y) + E(x|y)^2 x alpha. Alpha can be approximate using the score based t-test
reg zrs mu, nocons nohead //Beta is an approximation of alpha = .0237247
//Table 1
//Model Unadjusted for overdispersion
glm d ibn.end agep2-agep5 depr2-depr5, family(poisson) link(rs d_star) lnoffset(y) eform nocons nolog baselevels
eststo table1
//Model adjusted for overdispersion scaling SE
glm d ibn.end agep2-agep5 depr2-depr5, family(poisson) link(rs d_star) lnoffset(y) eform nocons nolog baselevels scale(x2)
eststo table2
//Model adjusted for overdispersion robust SE
glm d ibn.end agep2-agep5 depr2-depr5, family(poisson) link(rs d_star) lnoffset(y) eform nocons nolog baselevels vce(robust)
eststo table3
//Model adjusted for overdispersion using NBR (modelling mean and variance)
glm d ibn.end agep2-agep5 depr2-depr5, family(nbin .0237247) link(rs d_star) lnoffset(y) eform nocons nolog baselevels
eststo table4
estimates table table1 table2 table3 table4, stat(aic deviance df dispers_ps) b(%7.3f) se(%6.3f) p(%4.3f) eform
return list
esttab using table.tex, label ci nostar nodepvars brackets scalars(aic deviance df dispers_ps) ///
booktabs nomtitles ///
title("Piecewise exponential regression excess mortality models goodness of fit and point estimates (95$\%$CI)") eform replace
************************************************************************************************************************
*B) Flexible Poisson piecewise relative survival model with restricted cubic splines and time dependent effect of deprivation
************************************************************************************************************************
use England_breast_1997_2007.dta,clear
stset time, fail(dead=1) exit(time 10) id(ID2)
strs using popmort_England_2, ///
breaks(0(`=1/12')5) noshow ///
diagage(agediag) diagyear(yeardiag) ///
attage(age) attyear(year) ///
```

```
mergeby(ctry dep region sex year age) ///
by(agegroup dep) notables ///
survprob(survprob) ///
savgroup(group2.dta, replace)
//FLEXIBLE parametric piecewise exponential regression excess mortality model
use group2.dta, clear
gen midtime=(start + end)/2
gen lnmidtime=ln(midtime)
rcsgen lnmidtime, gen(rcs) df(3) fw(d) orthog
rcsgen lnmidtime, gen(rcstvc) df(3) fw(d) orthog
quietly tab agegroup, gen(agep)
quietly tabulate dep, gen(depr)
forvalues i= 2/5{
forvalues j= 1/3{
gen dep‘i’rcs‘j’ = depr‘i’*rcstvc‘j’
}
}
//Testing overdispersion
glm d rcs1-rcs3 agep2-agep5 dep?rcs? depr2-depr5, family(poisson) link(rs d_star) lnoffset(y) eform nolog baselevels
predict double mu, mu
//Score based t-test: H0 E(x|y) = Var(x|y)
gen double zrs =((d-mu)^2-d)/(mu)
reg zrs mu, nocons nohead
//Figure 2
//Flexible PEREM model allowing for the time dependent effect of deprivation without correcting for overdispersion
glm d rcs1-rcs3 dep?rcs? agep2-agep5 depr2-depr5, family(poisson) link(rs d_star) lnoffset(y) eform nolog baselevels
forvalues i = 2/5{
predictnl lhr‘i’ = _b[depr‘i’] + _b[agep‘i’] + _b[dep‘i’rcs1]*dep‘i’rcs1 + ///
    _b[dep‘i’rcs2]*dep‘i’rcs2 + _b[dep‘i’rcs3]*dep‘i’rcs3, ///
ci(lhr‘i’_lci lhr‘i’_uci)
gen hr‘i’=exp(lhr‘i’)
gen hr‘i’_lci=exp(lhr‘i’_lci)
gen hr‘i’_uci=exp(lhr‘i’_uci)
}
local title2 "Q2"
local title3 "Q3"
local title4 "Q4"
local title5 "Q5"
forvalues i = 2/5 {
twoway (rline hr‘i’_lci hr‘i’_uci midtime if depr‘i’ == 1 & agep‘i’==1) ///
(line hr‘i’ midtime if depr‘i’ == 1 & agep‘i’==1, lpattern(solid)) ///
(function y = 1, lpattern(dash) lwidth(thin) range(0 5)) ///
,yscale(log) name(hr‘i’, replace) ///
ylabel(0.5 1 2 4 8, format(%3.1f) angle(h)) ///
xtitle("Years from Diagnosis") ///
ytitle("Excess Mortality Rate Ratio") ///
legend(off) nodraw ///
title("Deprivation ‘title‘i’’") ///
plotregion(style(none))
}
graph combine hr2 hr3 hr4 hr5, ycommon nocopies name(Figure2A)
drop lhr2 lhr2_lci lhr2_uci hr2 hr2_lci hr2_uci lhr3 lhr3_lci lhr3_uci hr3 hr3_lci hr3_uci ///
lhr4 lhr4_lci lhr4_uci hr4 hr4_lci hr4_uci ///
lhr5 lhr5_lci lhr5_uci hr5 hr5_lci hr5_uci
//Flexible PEREM model allowing for the time dependent effect of deprivation and correcting for overdispersion using robust SE
glm d rcs1-rcs3 dep?rcs? agep2-agep5 depr2-depr5, family(poisson) link(rs d_star) lnoffset(y) eform nolog baselevels vce(robust)
forvalues i = 2/5{
predictnl lhr‘i’ = _b[depr‘i’] + _b[agep‘i’] + _b[dep‘i’rcs1]*dep‘i’rcs1 + ///
    _b[dep‘i’rcs2]*dep‘i’rcs2 + _b[dep‘i’rcs3]*dep‘i’rcs3, ///
ci(lhr‘i’_lci lhr‘i’_uci)
gen hr‘i’=exp(lhr‘i’)
gen hr‘i’_lci=exp(lhr‘i’_lci)
gen hr‘i’_uci=exp(lhr‘i’_uci)
}
local title2 "Q2"
local title3 "Q3"
local title4 "Q4"
local title5 "Q5"
forvalues i = 2/5 {
twoway (rline hr‘i’_lci hr‘i’_uci midtime if depr‘i’ == 1 & agep‘i’==1) ///
(line hr‘i’ midtime if depr‘i’ == 1 & agep‘i’==1, lpattern(solid)) ///
(function y = 1, lpattern(vsdash) lwidth(medium thick) range(0 5)) ///
,yscale(log) name(hr‘i’, replace) ///
ylabel(0.5 1 2 4 8, format(%3.1f) angle(h)) ///
xtitle("Years from Diagnosis") ///
ytitle("Excess Mortality Rate Ratio") ///
legend(off) nodraw ///
title("Deprivation ‘title‘i’’") ///
plotregion(style(none))
}
```

```
graph combine hr2 hr3 hr4 hr5, ycommon nocopies name(Figure2B)
graph combine Figure2A.gph Figure2B.gph
```
